# Pretreatment platelet count as a predictor for survival and distant metastasis in nasopharyngeal carcinoma patients

**DOI:** 10.3892/ol.2015.2872

**Published:** 2015-01-13

**Authors:** YU-PEI CHEN, CHEN CHEN, ZHUO-YAO MAI, JIN GAO, LU-JUN SHEN, BING-CHENG ZHAO, MENG-KUN CHEN, GANG CHEN, FANG YAN, TONG-YI HUANG, YUN-FEI XIA

**Affiliations:** 1 State Key Laboratory of Oncology in Southern China, Sun Yat-Sen University, Guangzhou, Guangdong, P.R. China; 2 Zhongshan School of Medicine, Sun Yat-Sen University, Guangzhou, Guangdong, P.R. China; 3 Department of Radiation Oncology, Anhui Provincial Hospital, Hefei, Anhui, P.R. China; 4 Department of Radiation Oncology, Cancer Center, Sun Yat-Sen University, Guangzhou, Guangdong, P.R. China

**Keywords:** platelet count, nasopharyngeal carcinoma, radiotherapy, concurrent chemoradiotherapy, predictor, prognosis

## Abstract

The aim of the present study was to investigate the prognostic value of different pretreatment platelet (PLT) counts on the treatment outcome in nasopharyngeal carcinoma (NPC) patients receiving concurrent chemoradiotherapy (CCRT) or radiotherapy (RT) alone. A total of 1,501 NPC patients, including 412 receiving CCRT and 1,089 receiving RT, were enrolled in the present study. The PLT count cut-off points for the CCRT and RT groups were 150 and 300×10^9^/l, respectively, and the PLT counts were categorized it into three groups: Low (PLT≤150×10^9^/l), moderate (150×10^9^/l<PLT≤300×10^9^/l) and high (PLT>300×10^9^/l). To identify independent predictors of overall survival (OS), the Cox proportional hazards model was used to determine local-regional recurrence-free survival (LRFS) and distant metastasis-free survival (DMFS) rates in the CCRT and RT patients. Furthermore, univariate and multivariate analysis indicated that compared with a moderate PLT count, a low PLT count was an independent unfavorable prognostic factor for OS rate in CCRT patients [hazard ratio (HR), 2.024; 95% confidence interval (CI), 1.165–3.516], and a high PLT count was an independent unfavorable prognostic factor for OS and DMFS rates in CCRT (OS: HR, 1.742; 95% CI, 1.090–2.786; DFMS: HR, 2.110; 95%CI, 1.084–4.108) and RT (OS: HR, 1.740; 95%CI, 1.283–2.362; DMFS: HR, 2.819; 95% CI, 1.766–4.497) patients. Compared with a low PLT count, a high PLT count was significantly and independently associated with a poor DMFS rate in the RT patients (P=0.025; HR, 2.454; 95% CI, 1.121–5.372). Therefore, the present study indicates that low and high PLT counts may be useful indicators of survival and distant metastasis in NPC patients who have undergone radiation treatment.

## Introduction

Nasopharyngeal carcinoma (NPC) is a unique type of head and neck cancer, which exhibits a distinct endemic distribution, with a particularly high incidence in Southern China and its surrounding regions (1). Currently, radiotherapy (RT) is the standard treatment modality for NPC, and advances in diagnostic imaging, radiotherapeutic techniques and chemotherapy regimens have improved the treatment outcomes of such patients (2,3). However, numerous trials investigating the adoption of RT and chemotherapy combination treatment strategies have not observed a reduced incidence of distant metastasis, therefore, distant metastasis continues to be an important reason for a poor prognosis in NPC patients (4,5).

Currently, the most important prognostic factor for NPC is the extent of the disease, which is defined using the tumor-node-metastasis staging system (6). However, a number of additional prognostic factors, which may significantly affect the prognosis, appear to be directly or indirectly associated with the extent of the NPC. Identification of these factors may provide novel indicators to aid in the improvement of the prognosis of NPC patients.

Platelets (PLTs) serve various roles in physiological and pathological pathways, and were initially associated with oncological processes, particularly the process of tumor metastasis, in the nineteenth century (7). Tumor-associated thrombocytosis is commonly observed in patients with solid cancer and has been identified as an unfavorable prognostic factor in numerous types of solid cancer, including oral squamous cell carcinoma (8), and esophageal (9), bronchial and lung (10), gastric (11) and breast (12) cancer. Furthermore, the percentage and the prognostic effect of an increased PLT count appears to vary depending on the disease and may change with the geological location (13). Thrombocytopenia may additionally be induced in patients with solid cancer, for example, by chemotherapy and the cancer itself (14). Previously, a decreased PLT count was identified as a prognostic factor for poor survival in esophageal cancer patients (15), and Schwarz and Keny (16) identified that a low preoperative PLT count was associated with poor survival following the resection of periampullary cancer; however, Domínguez *et al* (17) reported that a low PLT count was neither an adverse nor a favorable prognostic factor in resected pancreatic ductal adenocarcinoma. Thus, limited evidence is available to clarify the prognostic value of a decreased PLT count in other types of solid cancer.

PLT count may serve as a prognostic factor for cancer patients, however, there are few studies regarding its prognostic value in NPC. Our previous study identified that a PLT count of >300×10^9^/l prior to RT is a predictor of poor survival and distant metastasis in NPC patients (18); however, the prognostic value of a decreased PLT count was not considered. In addition, the administration of chemotherapeutic agents may have an impact on the PLT count via the inhibition of marrow function; thus, neoadjuvant chemotherapy may affect the PLT count prior to radiation treatment and the prognostic value of the PLT count may differ in patients receiving concurrent chemoradiotherapy (CCRT) (19). Therefore, in the present study, the sample size was enlarged compared with our previous study (18) and NPC patients receiving CCRT or RT alone were enrolled to investigate the prognostic significance of different pretreatment PLT counts.

## Patients and methods

### Patients

A retrospective review of 2,820 newly diagnosed NPC patients with no evidence of distant metastasis was conducted in the Sun Yat-Sen University Cancer Center (SYSUCC) between November 2000 and December 2004. The inclusion criteria were as follows: i) Newly diagnosed, histologically determined NPC; ii) no distant metastasis; and iii) currently receiving radical RT. The exclusion criteria were as follows: i) Treated with neoadjuvant or adjuvant chemotherapy (n=1,092); ii) loss of follow-up within five years (n=186); and iii) presence of concomitant diseases, which may affect PLT count, including inflammation, autoimmune disease, history of blood transfusion, liver cirrhosis, splenic disease and severe hypertension (n=41). Thus, a total of 1,501 NPC patients receiving CCRT or RT were enrolled in the present study. Computed tomography and/or magnetic resonance imaging were essential for disease staging prior to treatment, and all patients were restaged according to the 2009 American Joint Committee on Cancer staging system (20).

### PLT measurement and grouping

**Figure 1:**
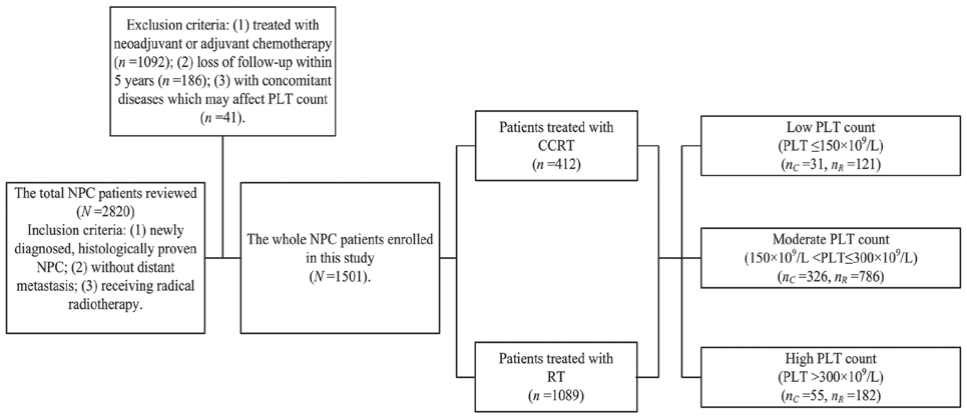
Flowchart of the study design. NPC, nasopharyngeal carcinoma; PLT, platelet; CCRT, concurrent chemoradiotherapy; RT, radiotherapy alone; n_C_, number of patients receiving concurrent chemoradiotherapy; n_R_, number of patients receiving radiotherapy alone.

Pretreatment PLT counts were measured at baseline within seven days of the commencement of RT for all patients. In accordance with a number of previous studies, including our previous study, a PLT count of >300×10^9^/l was considered to be of prognostic significance (18,21,22). A PLT count of <150×10^9^/l is associated with a poor treatment outcome in esophageal cancer patients (15) and indicates the requirement for a change in the chemotherapy administration pattern in solid cancers (14). Accordingly, the present study used 150 and 300×10^9^/l as the cut-off points and the PLT count was categorized it into three groups: Low (PLT≤150×10^9^/l), moderate (150×10^9^/l<PLT≤300×10^9^/l) and high (PLT>300×10^9^/l) (Fig. 1).

### RT

Definitive-intent RT with high energy 6–8 MV X-ray using a linear accelerator [Varian Clinac iX (Varian Medical Systems, Inc., Palo Alto, CA, USA), Elekta Precise (Elekta, Stockholm, Sweden) or Siemens Primus (Siemens Medical Solutions USA, Inc., Malvern, PA, USA)] was used to treat all the patients; 1,362 (90.7%) patients were treated with two-dimensional conformal RT (2D-CRT), 42 (2.8%) patients were treated with 3D-CRT and 97 (6.5%) patients were treated with intensity-modulated RT (IMRT).

Opposing lateral facial-cervical fields were used in the 2D-CRT to ensure that the nasopharynx and upper cervical lymphatic drainage region were targeted, and one lower anterior cervical field was used to cover the lower cervical region. Following radiation at a dose of 36–40 Gy, opposing lateral preauricular fields were used for the primary region and anterior split neck fields were used for the cervical region. Furthermore, the primary tumor was irradiated to a dose of 60–78 Gy. The irradiation dose for patients undergoing 2D-CRT was 50–54 Gy to the prophylactic areas; however, for 3D-CRT, the total prescribed dose was 66–72 Gy to the gross tumor volume of the nasopharynx (GTVnx), 60–70 Gy to the region involved by the metastatic lymph nodes (GTVnd), 60 Gy to the clinical target volume-1 (CTV-1), the GTVnx and an additional 5–10-mm margin, and 50–54 Gy to the prophylactic irradiating region (CTV-2). For IMRT, the target definition and delineation were the same as the aforementioned values for 3D-CRT. The prescription dose was 68 Gy to the GTVnx, 60–64 Gy to the GTVnd of the neck, 60 Gy to the CTV-1 and 54 Gy to the CTV-2.

### Chemotherapy

CCRT was received by 412 (27.4%) patients as cisplatin/carboplatin plus 5-fluorouracil (FU) or cisplatin alone. CCRT was mainly used for stage III–IV patients; the regimens for CCRT were mainly cisplatin alone. The cisplatin/carboplatin plus 5-FU regimen consisted of 70–100 mg/m^2^ cisplatin or 300–400 mg/m^2^ carboplatin on day one, plus 500–1000 mg/m^2^/day 5-FU on days 1–5 every 3–4 weeks for 2–3 cycles. By contrast, the cisplatin alone regimen consisted of 30–40 mg/m^2^ cisplatin every week for 6–7 cycles. The dose ranges were based on the conditions of the patients; if the side effects were severe, then the doses were reduced accordingly.

### Follow-up

Following the completion of treatment, patients were followed up every three months for the first three years, with the intervals gradually increasing to 6–12 months after three years. The follow-up data was last reviewed in February 2011. The assessed end-points included overall survival (OS), local-regional recurrence-free survival (LRFS) and distant metastasis-free survival (DMFS). OS was calculated as the time from the commencement of RT to mortality by any cause, and LRFS and DMFS were calculated as the time from the commencement of RT to the initial occurrence of local-regional or distant failure, respectively.

### Statistical analysis

All analyses were performed using SPSS software (version 19.0; IBM SPSS, Armonk, NY, USA). The χ^2^ test was used to compare categorical variables between the three PLT groups, and the rates of OS, LRFS and DMFS were estimated by means of the Kaplan-Meier method, and were compared between subgroups using the log-rank test. Multivariate analysis was performed by using the Cox proportional hazards model to analyze the independent significance of various variables in the CCRT and RT patients. Two-sided P-values of <0.05 were considered to indicate a statistically significant difference.

## Results

### Patient characteristics

The baseline characteristics of the 1,501 patients analyzed in the present study are shown in Table I. The median patient age was 46 years (range, 11–78 years). In total, 1,375 (91.6%) patients presented with undifferentiated non-keratinizing carcinoma, 117 (7.8%) with differentiated non-keratinizing carcinoma and nine (0.6%) with other types of NPC. The median duration of follow-up was 87 months (range, 2–125 months) and the pretreatment PLT count range was 58–600×10^9^/l, with a mean value of 232×10^9^/l. Furthermore, the PLT count groups were divided as follows: Low PLT count, 152 (10.1%) patients; moderate PLT count, 1,112 (74.1%) patients; and high PLT count, 237 (15.8%) patients.

Within the cohort, 412 (27.4%) patients received CCRT while 1,089 (72.6%) patients received RT. The pretreatment PLT count was significantly correlated with gender in the CCRT (P<0.001) and RT (P=0.044) patients (Table I), and overall, female patients exhibited a higher PLT count. Furthermore, no significant differences were identified in age, clinical stage, T stage and N stage between the CCRT or RT PLT groups.

### Treatment outcome of the PLT groups

Of the 1,501 patients in the present study, 250 (16.7%) developed local-regional failure, 132 (8.8%) developed distant metastasis and 366 (24.4%) succumbed. The five-year OS, LRFS and DMFS rates of the total cohort were 79.3, 84.9 and 91.6%, respectively.

Table II indicates the five-year OS, LRFS and DMFS rates of the three PLT groups in the CCRT and RT patients. Among the PLT groups, joint analysis identified significant differences in the OS and DMFS rates of patients administered CCRT (P=0.005 and P=0.036, respectively) and RT (P=0.005 and P<0.001, respectively; Table II). However, no significant differences in LRFS were identified among the PLT groups in the CCRT or RT patients (all P>0.05).

**Figure 2:**
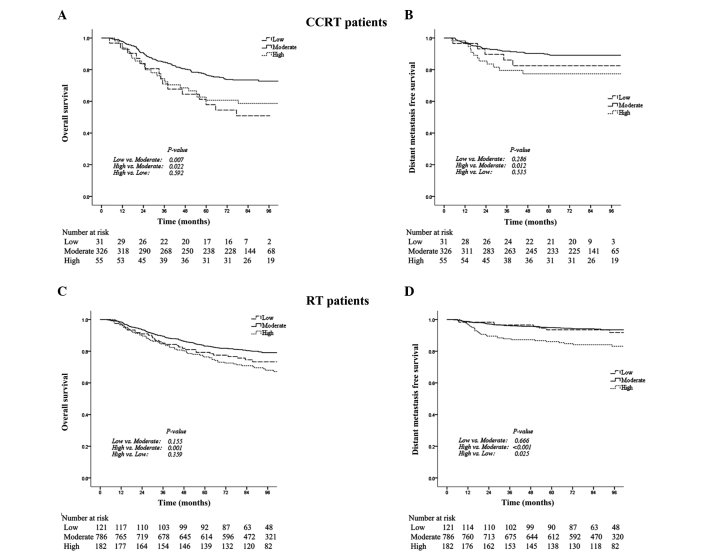
Kaplan-Meier curves indicating the overall survival (OS) and distant metastasis free survival (DMFS) rates in the low, moderate and high platelet (PLT) count groups, among patients receiving CCRT or RT. P-values were determined using the log-rank test for low vs. moderate, high vs. moderate and high vs. low PLT counts. In the CCRT patients, (A) the five-year OS rates in the low, moderate and high PLT groups were 57.9, 76.7 and 60.7% (P=0.007, P=0.022 and P=0.592), respectively and (B) the five-year DMFS rates in the low, moderate and high PLT groups were 82.5, 89.5 and 77.4% (P=0.286, P=0.012 and P=0.535), respectively. In the RT patients, (C) the five-year OS rates in the low, moderate and high PLT groups were 79.3, 83.2 and 76.4% (P=0.155, P=0.001 and P=0.359), respectively; and (D) the five-year DMFS rates in the low, moderate and high PLT groups were 93.5, 94.9 and 86.0% (P=0.666, P<0.001 and P=0.025), respectively. CCRT, concurrent chemoradiotherapy; RT, radiotherapy alone.

Additional analysis of the subgroups identified that in the CCRT patients, the five-year OS rate in the low PLT group was significantly lower compared with the moderate PLT group (56.9 vs. 76.7%; P=0.007; Table II; Fig. 2A). Furthermore, the five-year OS and DMFS rates in the high PLT group were significantly lower compared with the moderate PLT group (OS: 60.7 vs. 76.7%; P=0.022; DMFS: 77.4 vs. 89.5%; P=0.012; Table II; Fig. 2A and B).

In the RT patients, the OS and DMFS rates were not significantly different between the low and moderate PLT groups. However, the five-year OS and DMFS rates in the high PLT group were significantly lower than those in the moderate PLT group (OS: 76.4 vs. 83.2%; P=0.001; DMFS: 86.0 vs. 94.9%; P<0.001; Table II; Fig. 2C and D) and the five-year DMFS rate in the high PLT group was significantly lower than that in the low PLT group (86.0 vs. 93.5%; P=0.025; Table II; Fig. 2D).

### Prognostic significance of the PLT count

Table III summarizes the univariate and multivariate analyses of relevant prognostic factors in the CCRT and RT patients. Variables with P-values >0.10 were excluded from the model. Univariate analysis indicated that compared with a moderate PLT count, a low PLT count was a significant predictor for a poor OS rate in the CCRT patients only, while a high PLT count was a significant predictor for poor OS and DMFS rates in the CCRT and RT patients (Table III). Additionally, a high PLT count was significantly associated with a poor DMFS rate in the RT patients in comparison with a low PLT count (P=0.025).

**Figure 3:**
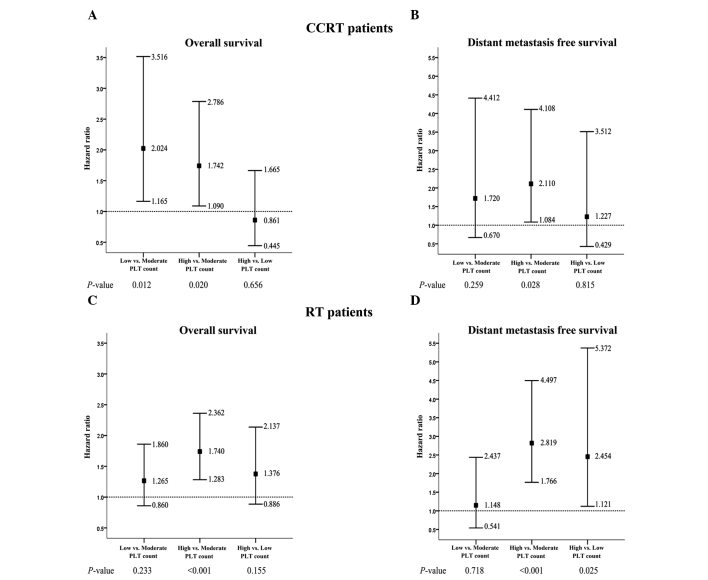
Hazard ratios and 95% confidence intervals of the PLT counts (low vs. moderate; high vs. moderate; high vs. low) for (A) overall survival (OS) rates in the CCRT patients; (B) distant metastasis-free survival (DMFS) rates in the CCRT patients; (C) OS rates in the RT patients; and (D) DMFS rates in the RT patients. CCRT, concurrent chemoradiotherapy; PLT, platelet; RT, radiotherapy alone.

Furthermore, multivariate analysis identified that in CCRT patients, a low PLT count was an independent negative prognostic factor for OS rate [hazard ratio (HR), 2.024; 95% confidence interval (CI), 1.165–3.516] and a high PLT count was an independent negative prognostic factor for OS (HR, 1.742; 95% CI, 1.090–2.786) and DMFS (HR, 2.110; 95% CI, 1.084–4.108) rate compared with a moderate PLT count (Table III; Fig. 3A and B). Furthermore, in terms of OS rate, the negative effect of a low PLT count appeared to be greater than the negative effect of a high PLT count (Fig. 3A).

In the RT patients, a low PLT count was not determined to be a prognostic factor for OS or DMFS rate in comparison with a moderate PLT count; however, a high PLT count was identified to be an independent negative prognostic factor for OS (HR, 1.740; 95% CI, 1.283–2.362) and DMFS (HR, 2.819; 95% CI, 1.766–4.497; Table III; Fig. 3C and D) rate in comparison with a moderate PLT count. In addition, a high PLT count was independently associated with a poor DMFS rate compared with a low PLT count (HR, 2.454; 95% CI, 1.121–5.372; Table III; Fig. 3D).

## Discussion

The present study evaluated the prognostic value of low, moderate and high pretreatment PLT counts in NPC patients. The study demonstrated that in comparison to a moderate PLT count, a low PLT count was significantly and independently associated with a poor OS rate in CCRT patients, and a high PLT count was significantly and independently associated with poor OS and DMFS rates in CCRT and RT patients. In CCRT patients, this negative effect on OS rate was greater in the presence of a low PLT count compared with a high PLT count. Furthermore, compared with a low PLT count, a high PLT count was significantly and independently associated with a poor DMFS rate in RT patients. These observations highlight the importance of determining the pretreatment PLT count and to the best of our knowledge, represents the first study to address the prognostic value of different pretreatment PLT count levels in NPC patients who have undergone radiation treatment.

In cancer patients, the administration of cytotoxic agent chemotherapy is a common reason for a decreased PLT count, while disseminated intravascular coagulation, which exhibits more chronic and subclinical properties, is the most common non-iatrogenic cause of a reduced PLT count (24,25). Alidina *et al* (15) identified that a PLT count of <150×10^9^/l was associated with poor survival in esophageal cancer (HR, 6.58; P=0.001). These results are consistent with those of the present study, which demonstrated that a low PLT count was an unfavorable prognostic factor for OS in CCRT patients when compared with a moderate PLT count. Furthermore, the negative effect of a low PLT count was greater than that of a high PLT count. However, in comparison to a high PLT count, a low PLT count was significantly associated with reduced metastasis in RT but not CCRT patients, which may be explained by the role of PLTs in tumor metastasis. A large number of studies have indicated that an increased PLT count may affect the metastatic potential of tumor cells by facilitating immune evasion, promoting extravasation and impeding natural killer cells (7,24). However, in CCRT patients in the present study, the association between low or high PLT count and DMFS was not significant, and a low PLT count was significantly associated with a poor OS rate compared with a moderate PLT count, despite exhibiting a greater negative effect on OS than a high PLT count. This phenomenon indicates that the negative effect of a low PLT count may only becomes apparent in CCRT patients. Furthermore, Schwarz (26) proposed that a decreased PLT count may reflect a poor performance status with megakaryocyte inhibition in pancreatic cancer. In addition, a low PLT count may contribute to an increase in the risk of developing hemorrhagic complications (27), invasive infection and chemotherapy intolerance (28), which all result in a poor prognosis. Therefore, the present study proposes that a decreased PLT count has a more apparent negative effect in patients receiving CCRT, as CCRT may be tolerated less well than RT, resulting in the development of more profound myelosuppression and causing patients to become susceptible to complications associated with abnormal coagulation, which ultimately results in inferior treatment outcomes.

The cause of tumor-associated thrombocytosis remains unclear, however, the tumor-associated production of granulocyte-macrophage colony-stimulating factor or thrombopoietin (TPO) mediated by interleukin-6 is considered to be responsible for the increase in PLT count observed in cancer patients (29). In the present study, a high PLT count was clearly demonstrated to be an unfavorable prognostic factor for OS and DMFS rate in the CCRT and RT patients compared with a moderate PLT count. As well as the aforementioned role of PLTs in tumor metastasis, the possible roles of PLTs in tumor growth and angiogenesis may explain this unfavorable prognostic effect; for example, PLTs are able to secrete a number of proangiogenic cytokines, including vascular endothelial growth factor (30) and thymidine phosphorylase (31). These PLT-derived factors can affect hemostasis, as well as proliferative and angiogenic activity, which may be associated with the depth of tumor invasion and a poor response to CRT (32,33). In addition to these proangiogenic cytokines, PLTs may promote angiogenesis directly via integrins, which mediate cell-to-cell adhesion (34). Furthermore, the development of a hypercoagulable state in cancer patients can increase the risk of thrombosis (35), and chemotherapy may additionally potentiate this risk via endothelial cell damage, stimulation of PLT aggregation and a reduction in anticoagulant synthesis (36). Therefore, the present study proposes that chemotherapy-associated thrombophilia may be an important explanation for the poor outcome of the CCRT patients with high PLT counts observed in the present study. This hypothesis is supported by a previous lung cancer study conducted by Zecchina *et al* (37), in which thrombocytosis at the time of chemotherapy administration was found to be involved in triggering thrombotic complications.

As the pretreatment PLT count appears to be an independent prognostic factor affecting NPC treatment outcome, corresponding active treatments should be considered prospectively. For patients with a low PLT count, particularly those receiving chemotherapy, PLT transfusion is a rapid and effective means of controlling bleeding (38), however, it is costly, may transfer infection and specific patients may develop an immunoreaction or become refractory to the treatment strategy. Thus, an alternative treatment strategy is TPO, which can be administered in combination with chemotherapeutic agents to prevent the occurrence of severe thrombocytopenia (19). Yang *et al* (39) reported that the use of uninterrupted TPO support for the treatment of two cases of NPC with thrombocytopenia was well-tolerated, and oprelvekin was identified to be effective in the treatment of solid cancer patients with chemotherapy-induced thrombocytopenia (40). However, these treatments strategies may have an inherent oncological risk due to the aforementioned pro-tumor effects of PLT; therefore, satisfactory optimization of the therapeutic strategy is required. With respect to thrombocytosis, anticoagulants have been successfully employed in animal models to inhibit tumor metastasis and tumor-associated thrombosis (41,42). A meta-analysis of 11 studies demonstrated that anticoagulants significantly improved the OS rate in cancer patients, despite increasing the risk of bleeding complications (43); however, anticoagulants lack selectivity, which affects hemostasis.

The retrospective nature of the present study and the lack of detailed data collected regarding patient complications following RT impedes further interpretation of the prognostic value of pretreatment PLT counts. However, to the best of our knowledge, the present study is the first to report the prognostic effect of different pretreatment PLT count levels in NPC patients following radiation treatment, and the first to propose its clinical significance. In conclusion, the pretherapeutic period provides a good opportunity to modify the treatment strategy of NPC patients for an improved prognosis. Thus, pretreatment PLT count, which can be easily and cheaply determined, represents an important therapeutic tool in NPC. Additional studies are required to clarify the effect of PLT levels in cancer and the benefits of corresponding therapeutic strategies.

## Figures and Tables

**Table I:** Baseline characterictics in the 1,501 nasopharyngeal carcinoma patients.

		CCRT patient PLT count (n=412)				RT patient PLT count (n=1089)			
					
Patient characteristic	Total patients, n (%)(n=1501)	Low, n (%)	Moderate, n (%)	High, n (%)	P-value^a^	Low, n (%)	Moderate, n (%)	High, n (%)	P-value^a^
Age, years					0.326				0.289
≤45	734 (48.9)	13 (41.9)	163 (50.0)	32 (58.2)		52 (43.0)	379 (48.2)	95 (52.2)	
>45	767 (51.1)	18 (58.1)	163 (50.0)	23 (41.8)		69 (57.0)	407 (51.8)	87 (47.8)	
Gender					0.044				<0.001
Male	1158 (77.1)	26 (83.9)	271 (83.1)	38 (69.1)		99 (81.8)	613 (78.0)	111 (61.0)	
Female	343 (22.9)	5 (16.1)	55 (16.9)	17 (30.9)		22 (18.2)	173 (22.0)	71 (39.0)	
Clinical stage^b^					0.237				0.866
I	127 (8.5)	2 (6.5)	11 (3.4)	2 (3.6)		10 (8.3)	85 (10.8)	17 (9.3)	
II	583 (38.8)	5 (16.1)	93 (28.5)	12 (21.8)		53 (43.8)	344 (43.8)	76 (41.8)	
III	543 (36.2)	17 (54.8)	155 (47.5)	22 (40.0)		43 (35.5)	247 (31.4)	59 (32.4)	
IV	248 (16.5)	7 (22.6)	67 (20.6)	19 (34.5)		15 (12.4)	110 (14.0)	30 (16.5)	
T stage					0.101				0.619
T1	326 (21.7)	5 (16.1)	59 (18.1)	7 (12.7)		24 (19.8)	191 (24.3)	40 (22.0)	
T2	600 (40.0)	12 (38.7)	112 (34.4)	14 (25.5)		56 (46.3)	333 (42.4)	73 (40.1)	
T3	360 (24.0)	9 (29.0)	101 (31.0)	15 (27.3)		26 (21.5)	170 (21.6)	39 (21.4)	
T4	215 (14.3)	5 (16.1)	54 (16.6)	19 (34.5)		15 (12,4)	92 (11.7)	30 (16.5)	
N stage					0.134				0.111
N0	466 (31.0)	10 (32.3)	52 (16.0)	13 (23.6)		49 (40.5)	283 (36.0)	59 (32.4)	
N1	620 (41.3)	8 (25.8)	145 (44.5)	20 (36.4)		45 (37.2)	323 (41.1)	79 (43.4)	
N2	376 (25.0)	10 (32.3)	114 (35.0)	20 (36.4)		27 (22.3)	161 (20.5)	44 (24.2)	
N3	39 (2.6)	3 (9.7)	15 (4.6)	2 (3.6)		0 (0.0)	19 (2.4)	0 (0.0)	
Treatment									
CCRT	412 (27.4)	31 (100.0)	326 (100.0)	55 (100.0)		0 (0.0)	0 (0.0)	0 (0.0)	
RT	1089 (72.6)	0 (0.0)	0 (0.0)	0 (0.0)		121 (100.0)	786 (100.0)	182 (100.0)	

a A χ^2^ test was performed to compare patient characteristics between the three PLT groups.

b According to the seventh edition of the AJCC/UICC staging system (23).

CCRT, concurrent chemoradiotherapy; PLT, platelet; RT, radiotherapy alone; T, tumor; N, node.

**Table II:** Treatment outcome of the three platelet groups in the 1,501 nasopharyngeal carcinoma patients.

Platelet count	Five-year OS, %	P-value^a^	Five-year LRFS, %	P-value^a^	Five-year DMFS, %	P-value^a^
CCRT patients (n=412)		0.005		0.153		0.036
Low	57.9		76.0		82.5	
Moderate	76.7		83.4		89.5	
High	60.7		72.4		77.4	
RT patients (n=1089)		0.005		0.224		<0.001
Low	79.3		81.8		93.5	
Moderate	83.2		86.9		94.9	
High	76.4		85.5		86.0	

a Determined by joint analysis.

OS, overall survival; LRFS, local-regional recurrence-free survival; DMFS, distant metastasis-free survival; CCRT, concurrent chemoradiotherapy; RT, radiotherapy alone.

**Table III:** Univariate and multivariate analyses of prognostic factors in the 1,501 nasopharyngeal carcinoma patients.

		Univariate	Multivariate^a^		
			
Treatment outcome	Variable	P-value	HR	95% CI	P-value
CCRT patients (n=412)					
Overall survival	Age (>45 vs. ≤45 years)	0.033	1.576	1.096–2.264	0.014
	N status (N2–3 vs. N0–1)	0.079	1.508	1.055–2.154	0.024
	PLT count				
	Low vs. moderate	0.026	2.024	1.165–3.516	0.012
	High vs. moderate	0.022	1.742	1.090–2.786	0.020
	High vs. low	0.857	0.861	0.445–1.665	0.656
Local-regional recurrence-free survival	Age (>45 vs. ≤45 years)	0.057	1.550	0.983–2.444	0.059
Distant metastasis-free survival	T status (T3–4 vs. T1–2)	0.069	1.616	0.912–2.863	0.100
	N status (N2–3 vs. N0–1)	0.092	1.627	0.933–2.835	0.086
	PLT count				
	Low vs. moderate	0.286	1.720	0.670–4.412	0.259
	High vs. moderate	0.012	2.110	1.084–4.108	0.028
	High vs. low	0.535	1.227	0.429–3.512	0.815
RT patients (n=1089)					
Overall survival	Age (>45 vs. ≤45 years)	0.001	1.667	1.286–2.161	<0.001
	Gender (female vs. male)	0.046	1.397	1.013–1.925	0.041
	N status (N2–3 vs. N0–1)	0.003	1.618	1.227–2.135	0.001
	PLT count				
	Low vs. moderate	0.149	1.265	0.860–1.860	0.233
	High vs. moderate	0.038	1.740	1.283–2.362	<0.001
	High vs. low PLT	0.822	1.376	0.886–2.137	0.155
Local-regional recurrence-free survival	Age (>45 vs. ≤45 years)	0.003	1.602	1.181–2.173	0.002
	N status (N2–3 vs. N0–1)	0.061	1.422	1.014–1.994	0.041
Distant metastasis-free survival	Age (>45 vs. ≤45 years)	0.083	1.553	0.998–2.416	0.051
	N status (N2–3 vs. N0–1)	0.035	1.653	1.032–2.648	0.037
	PLT count				
	Low vs. moderate	0.666	1.148	0.541–2.437	0.718
	High vs. moderate	<0.001	2.819	1.766–4.497	<0.001
	High vs. low PLT	0.025	2.454	1.121–5.372	0.025

a The following parameters were included in the multivariate analysis using Cox proportional hazards model by backward elimination: Age (>45 vs. ≤45 years); gender (female vs. male); T status (T3–4 vs. T1–2); N status (N2–3 vs. N0–1); PLT count (low vs. moderate; high vs. moderate; high vs. low). Variables with P-values >0.10 were excluded from the model.

HR, hazard ratio; CI, confidence interval; CCRT, concurrent chemoradiotherapy; N, node; PLT, platelet; T, tumor; RT, radiotherapy alone.
